# Two New Species of the Genus *Diderma* (Physarales, *Didymiaceae*) in China with an Addition to the Distribution

**DOI:** 10.3390/jof10080514

**Published:** 2024-07-23

**Authors:** Xuefei Li, Yonglan Tuo, You Li, Jiajun Hu, Frederick Leo Sossah, Dan Dai, Minghao Liu, Yanfang Guo, Bo Zhang, Xiao Li, Yu Li

**Affiliations:** 1 Engineering Research Centre of Edible and Medicinal Fungi, Ministry of Education, Jilin Agricultural University, Changchun 130118, China; lixuefei2020@163.com (X.L.); tuoyonglan66@163.com (Y.T.); 17790099278@163.com (Y.L.); hujjfungi@163.com (J.H.); flsossah@gmail.com (F.L.S.); m13082447311@163.com (D.D.); minghaoliu14@163.com (M.L.); 2 College of Mycology, Jilin Agricultural University, Changchun 130118, China; 3 School of Life Science, Zhejiang Normal University, Jinhua 321004, China; 4 Coconut Research Programme, Oil Palm Research Institute, Council for Scientific and Industrial Research (CSIR), Sekondi P.O. Box 245, Ghana; 5 Institute of Agricultural Applied Microbiology, Jiangxi Academy of Agricultural Sciences, Nanchang 330200, China; 6 Westerdijk Fungal Biodiversity Institute, Uppsalalaan 8, 3584 CT Utrecht, The Netherlands; y.guo@wi.knaw.nl

**Keywords:** new species, taxonomy, diversity, morphology, phylogeny

## Abstract

Myxomycetes are an important component of terrestrial ecosystems, and in order to understand their diversity and phylogenetic relationships, taxonomic issues need to be addressed. In our 1985–2021 biodiversity investigations in Shaanxi Province, Jilin Province, the Inner Mongolia Autonomous Region, Hubei Province, and Henan Province, China, *Diderma* samples were observed on rotten leaves, rotten branches, and dead wood. The samples were studied, based on morphological features coupled with multigene phylogenetic analyses of nSSU, EF-1α, and COI sequence data, which revealed two new species (*Diderma shaanxiense* sp. nov. and *D. clavatocolumellum* sp. nov.) and two known species (*D. radiatum* and *D. globosum*). In addition, *D. radiatum* and *D. globosum* were newly recorded in Henan Province and the Inner Mongolia Autonomous Region, respectively. The paper includes comprehensive descriptions, detailed micrographs, and the outcomes of phylogenetic analyses for both the newly discovered and known species. Additionally, it offers morpho-logical comparisons between the new species and similar ones.

## 1. Introduction

Myxomycetes are a unique group of small eukaryotic organisms found in a wide range of terrestrial ecosystems. They are one of the few protist groups that exhibit sufficient morphological characteristics to enable the application of the morphological species concept. These organisms belong to the Eumycetozoa within the Amoebozoa [1,2].

The genus *Diderma* was established by Persoon in the 18th century (1794) to accommodate species characterized by the absence of lime knots connecting the capillitium, dark brown or black spores in mass, and a peridium with two or three layers covered by calcareous or cartilaginous material [3,4]. *Diderma globosum* Pers. was designated as the type species [3,4,5,6,7,8]. Rostafinski placed the genus *Diderma* in the family *Didymiaceae* in 1873 [9,10], a classification adopted by subsequent mycologists [3]. Historically, this genus has been primarily identified based on morphological characteristics.

In recent years, the advancement of molecular biology has enabled researchers to conduct phylogenetic studies on the genus *Diderma* using sequences such as SSU and EF-1A [6,7,8]. These studies, which also utilized COI sequences, have demonstrated that *Diderma* forms a monophyletic clade within the family *Didymiaceae*.

Currently, over 1100 species of myxomycetes are known [11] but only 87 species of *Diderma* have been identified worldwide, with 30 species recorded in China [12,13,14,15,16]. In this study, we investigate the species diversity of *Diderma* and report the first occurrences of two species, *D*. *shaanxiense* and *D*. *clavatocolumellum*. Additionally, we document two new provincial records: *D*. *globosum* in the Inner Mongolia Autonomous Region and *D*. *radiatum* in Henan Province. We provide a morphological description of the new species.

## 2. Materials and Methods

### 2.1. Sampling and Morphological Studies

Habitat details of fresh myxomycetes were recorded at the time of collection. To prevent mixing or crushing, each specimen was wrapped in a single box. Fresh specimens were air-dried in a cool place shortly after returning from the field. Both macroscopic and micromorphological descriptions were based on dried materials. The color terminology used in our descriptions follows the Flora of British Fungi: Colour Identification Chart [17].

Macroscopic characteristics were observed using a Zeiss dissecting microscope (Axio Zoom V16, Carl Zeiss Microscopy GmbH, Göttingen, Germany), and photographs were taken with a Leica stereoscopic dissector (Leica M165, Wetzlar, Germany). The prepared specimens were observed using a Lab A1 microscope (Carl Zeiss AG, Jena, Germany) and documented with photographs using ZEN 2.3 software (Carl Zeiss AG).

Twenty mature spores from each species were measured in side view. Spore dimensions are presented as (a) b–c (d), where the b–c range encompasses at least 90% of the measured values, while “a” represents the smallest length and “d” represents the largest value, if present. Additionally, specimens for scanning electron microscopy (SEM) were mounted on copper stubs with double-sided tape or a thin layer of nail polish, sputter-coated with gold, and then observed and photographed using a Hitachi S-4800 SEM (Hitachi, Tokyo, Japan) to examine the ultrastructure of spores and capillitium. The examined collections have been deposited in the Herbarium of Mycology at Jilin Agriculture University (HMJAU), Jilin, China.

### 2.2. DNA Extraction, PCR Amplification, and Sequencing

DNA extraction from dried sporocarps was performed using the TIANamp Micro DNA Kit (TianGen Biotech Co., Ltd., Beijing, China) according to the manufacturer’s instructions. The polymerase chain reaction (PCR) mixture consisted of 1 μL (10 mmol/L) of each primer, 3 μL of DNA template, 12.5 μL of 2X SanTaq PCR Mix (Sangon Biotech, Shanghai, China), and water added to a final volume of 25 μL. The primers utilized for amplifying the small subunit ribosomal RNA (nSSU), translation elongation factor 1-alpha (EF-1α), and mitochondrial cytochrome c oxidase subunit I (COI) were SSU101F/P1R [18], PB1F/PB1R [19], and COMF/COMRs [20,21,22], respectively.

The PCR programs for amplifying the nSSU region were as follows: initial pre-denaturation at 95 °C for 6 min, followed by 32 cycles of denaturation at 94 °C for 1 min, annealing at 52 °C for 1 min, an extension at 72 °C for 1 min, and a final extension at 72 °C for 10 min. For the amplification of EF-1α, the PCR program included an initial pre-denaturation at 95 °C for 5 min, followed by 36 cycles of denaturation at 95 °C for 30 s, annealing at 65.4 °C for 30 s, extension at 72 °C for 1 min, and a final extension at 72 °C for 10 min. Similarly, for COI, the PCR parameters consisted of an initial pre-denaturation at 95 °C for 5 min, followed by 36 cycles of denaturation at 95 °C for 30 s, annealing at 52 °C for 20 s, extension at 72 °C for 1 min, and a final extension at 72 °C for 10 min. The PCR products were sequenced using the Sanger method by Kumei Biotechnology Co., Ltd. (Changchun, China).

### 2.3. Data Analysis

The sequences from different loci were auto-strategy aligned using MAFFT 7.490 software [23] and manually edited with BioEdit 7.1.3 and Clustal X 1.81 [24,25] separately. Separate phylogenetic analyses were conducted for each marker gene, and the resulting topologies were compared to assess congruence through visual inspection. The partition homogeneity test was then performed using PAUP 4.0 [26] to evaluate the compatibility of the marker sequences. Based on the test results, which indicated sufficient congruence, the marker sequences were concatenated for the final phylogenetic analysis. Both the Bayesian Inference (BI) and maximum likelihood (ML) methods were employed for constructing phylogenetic trees. The best-fit substitution models were determined using Modelfinder [27]. For maximum likelihood (ML) analyses, the datasets we were subjected to analysis using IQTREE 1.6.12 software [28] with a rapid bootstrap comprising 1000 bootstrap (BS) replicates. For the Bayesian analysis, posterior probabilities (PPs) were computed using Markov Chain Monte Carlo sampling (MCMC) in MrBayes on XSEDE v. 3.2.7a [29,30], employing the estimated evolutionary models. Runs of 4,000,000 generations were conducted, with trees sampled every 1000 generations, using eight heated and one cold Markov chain(s), with the initial 25% of samples discarded as burn-in. The average standard deviation of split frequencies was 0.003222, indicating a significant difference. If the average standard deviation of split frequencies was above 0.01, ESS values were checked using Tracer v1.7.1 software [31], where values greater than 200 indicated convergence. The final trees were visualized using ITOL version 6.9 [32]. Detailed information regarding reference strains is provided in Table 1 and Table S1.

## 3. Results

### 3.1. Phylogenetic Analysis

The topologies of individual marker sequences were examined through separate phylogenetic analyses for SSU, EF-1A, and COI genes. A visual inspection showed general congruence among the topologies. To quantitatively assess this congruence, a partition homogeneity test was performed using PAUP*. The results for the individual marker genes and the concatenated dataset are shown in Table S2.

The combined SSU-EF1A-COI dataset consisted of 88 nSSU, 70 EF-1α, and 16 COI sequences derived from 42 taxa representing 10 genera across three orders. This dataset encompassed 13 described species and 2 undescribed species of *Diderma*, with two sequences from *Echinostelium* serving as outgroups. In total, 18 new sequences were generated in this study, comprising 7 nSSU, 5 EF-1α, and 6 COI sequences (see Table 1). The final dataset comprised 6442 bp (including gaps), with 3980 bp (including gaps) from SSU, 1676 bp from EF-1α, and 786 bp from COI. The alignment was submitted to TreeBASE (http://purl.org/phylo/treebase/phylows/study/TB2:S31522, accessed on 15 July 2024).

**Figure 1:**
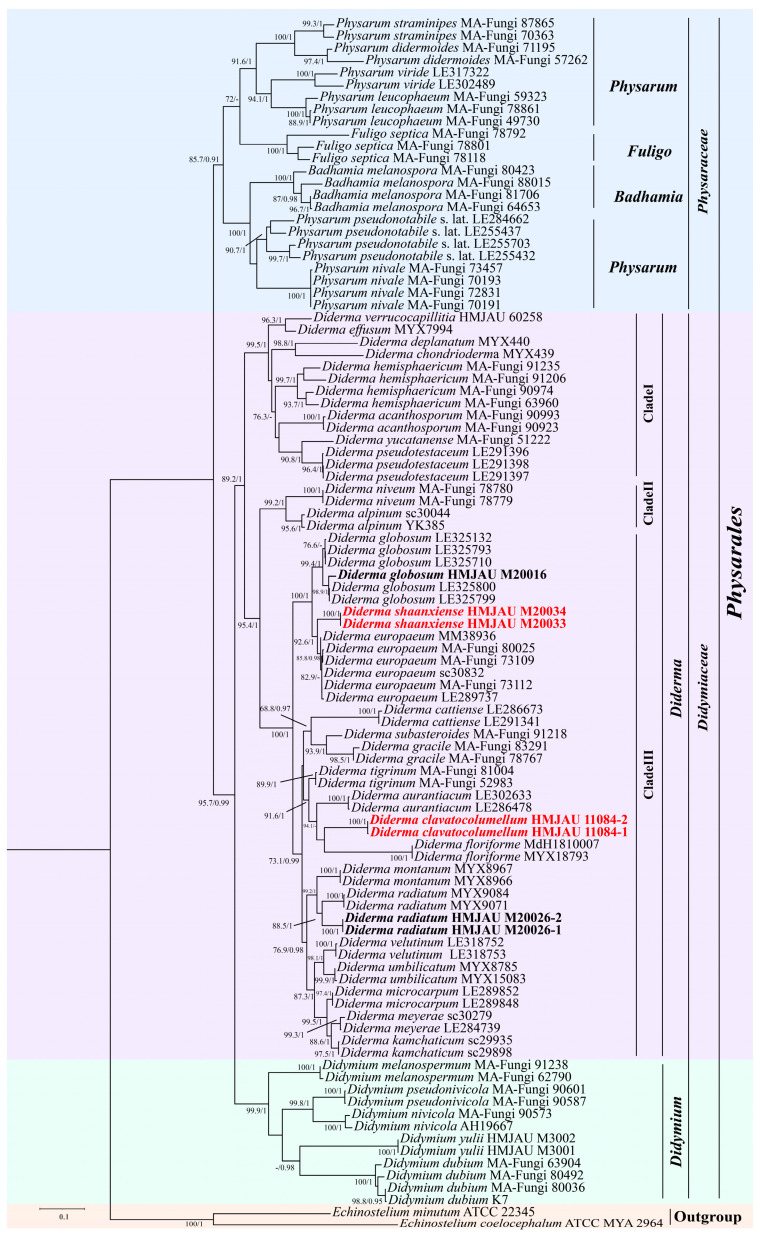
Maximum likelihood phylogenetic tree generated from the combined nSSU, EF-1α, and COI dataset of the *Diderma* species. The display on internal nodes indicates maximum likelihood bootstrap (BP) and Bayesian posterior probability (BPPS) values. The study species are marked in bold (known species) and red (new species) font for easy identification. (Notes: the final dataset comprised 6442 bp, including gaps, with 3980 bp (including gaps) from SSU, 1676 bp from EF-1α, and 786 bp from COI.).

The best nucleotide substitution models for the BI phylogeny were GTR+F+G4 for SSU and GTR+F+I+G4 for both EF-1α and COI. The Bayesian analysis ran for two million generations, yielding an average standard deviation of split frequencies of 0.003873. Subsequently, the same dataset and alignment underwent analysis using the ML method. In the ML analysis, the best nucleotide substitution model for SSU and EF-1A is TIM2e+I+G4, while the COI model is TIM2e+F+I+G4. A comparison of the overall topologies between the ML and BI trees indicated nearly identical results across all datasets. Given this congruence, the ML analysis was chosen as the representative phylogeny. Internal nodes of the tree are labeled with values representing ML and BI support. Branches display bootstrap values (BP) of ≥70% from the ML analysis and Bayesian posterior probabilities (BPPS) of ≥0.90 (Figure 1). The phylogenetic tree obtained revealed two distinct clades within *Didymiaceae*, representing *Diderma* and *Didymium* (Figure 1). Additionally, our findings revealed the division of the genus *Diderma* into three distinct clades. Among these, four sampled specimens constituted two separate clades indicative of the presence of two new species, namely *D*. *shaanxiense* and *D*. *clavatocolumellum*.

*Diderma shaanxiense* sequences formed a distinct branch with 100/1 support, closely related to *D*. *globosum* and *D*. *europaeum*. The phylogenetic distance between *D. shaanxiense* and *D. floriforme* must be very similar to that of *D. aurantiacum* and lower than that of *D. tigrinum*. These data indicate significant differences between them, supporting the classification of these two as independent species. Additionally, two sampled specimens, which clustered with *D*. *radiatum* and *D*. *globosum* with strong support, were confirmed as new records from Henan Province and the Inner Mongolia Autonomous Region, respectively.

### 3.2. Taxonomy

*Diderma shaanxiense* X.F. Li, B. Zhang and Y. Li, sp. nov.

MycoBank: MB 852952

Figure 2

Diagnosis. Columella hemispherical, with a shining layer of film. 

Holotype. China, Shaanxi Province, Baoji City, Meixian County, and Taibai Mountain National Forest Park National Forest Park; on the rotten leaves, 22 July 2014, B. Zhang, HMJAU M20033.

Etymology. The epithet “*shaanxiense*” refers to Shaanxi, the location of the holotype.

Description. Sporangia, sessile, spheroid, gregarious, mutual squeezing deformation. Peridium is double-layered; the outer layer is calcareous, white, smooth; the inner layer is membranous, grey, iridescent, sometimes wrinkled, and separated from the outer layer. Columella is developed, yellowish brown or white, calcareous, pulvinate, or hemispherical, with a shining layer of film. Hypothallus is inconspicuous. Capillitium is crude, branched, and anastomosing, with membranous enlargement, brown or colorless, occasionally with some darker thickenings. Spore-mass dark or dark brown, fawn under transmitted light (TL), 8–11 μm in diam., with warts. 

Habitat. On the rotten leaves.

Distribution in China. Shaanxi Province.

Distribution in the world. China.

Additional specimens examined. China, Shaanxi Province, Baoji City, Meixian County, Taibai Mountain National Forest Park National Forest Park, on the rotten leaves, 22 July 2014, B. Zhang, HMJAU M20034, HMJAU M20050; China, Shaanxi Province, Niubeiliang National Nature Reserve, on the rotten leaves, 21 July 2014, B. Zhang, HMJAU M20051.

Notes. *Diderma shaanxiense* is characterized by its shining layer of film columella. *Diderma shaanxiense* shares morphological similarities with *D. europaeum* and *D. globosum* in terms of sessile and subglobose sporocarpic, two-layered peridium. However, it can be differentiated by its iridescent columella, spores with warts, and small size (8–11 μm) compared to *D. europaeum*, while *D. europaeum* has a pulvinate columella, spores with spines, and larger size (10–12 μm). *D. shaanxiense* can be distinguished from *D. globosum* based on its small and pulvinate shining columella. From the phylogenetic tree, we can see that *D. shaanxiense* is located between *D. globosum* and *D. europaeum*, and a separate branch is formed.

*Diderma clavatocolumellum* X.F. Li, B. Zhang and Y. Li, sp. nov. 

MycoBank: MB 852955

Figure 3

Diagnosis. Columella club-shaped, developed, with yellow-brown stalk.

Holotype. China, Jilin Province, Yanji Prefecture, Antu County, Erdaobaihe, on the dead woods, 5 October 2001, H.Z. Liu and Tolgor, HMJAU 11084.

Etymology. The epithet “*clavatocolumellum*” refers to the club-shaped columella.

Description. Sporophores sporocarpic, gregaria, sessile or short-stalk, oblate or hemispherical, grey. Peridium is two-layered; the outer layer is cartilaginous, covered with a layer of lime granules, crustose, white, light brown and light ochre, smooth; the inner layer is tightly attached together with the outer layer, calcareous, white or grey. Irregular dehiscence at the top, basal petaloid dehiscence and eversion. Columella present, either developed or conspicuous, clavate, close to the top of the columella, rough, ochre-orange or ivory, 0.5–0.7 mm. Hypothallus is inconspicuous, membranous, translucent, brown. Capillitium is abundant, radiantly extending from the columella, brown or colorless, branching and connecting, with many dark brown swollen knots. Spores dark in mass, purple-brown via transmitted light, subglobose or oval, 9–11 (11.5) μm in diam., with obvious warts, sparse.

Habitat. On the dead woods.

Distribution in China. Jilin Province.

Distribution in the world. China.

Additional specimens examined. China, Jilin Province, Yanji Prefecture, Antu County, Erdaobaihe, on the dead woods, 5 October 2001, H.Z. Liu and Tolgor, HMJAU M20120, HMJAU M20121.

Notes. Morphologically, *D. clavatocolumellum* is similar to *D. alpinum* (Meyl.) Meyl. and *D. floriforme* with its double-layer peridium, spore size, and capillitium having swelling. However, *D. clavatocolumellum* can be distinguished from *D. alpinum* based on its developed and conspicuous columella (clavate), two layers of peridium that are tightly connected, membranous and brown hypothallus, and spores with warts; meanwhile, *D. alpinum* has a pulvinate columella; two layers of peridium kept away; easily separated, calcareous, and white hypothallus; and spores with spines. *Diderma clavatocolumellum* can be distinguished from *D. floriforme* based on its sessile or short stalk, while *D. floriforme* has a long stalk. From the phylogenetic tree, we can clearly see that *D. clavatocumullum* is related to *D*. *floriforme* and they are similar, but the genetic distance between the two is relatively far.

*Diderma globosum* Pers., Neues Mag. Bot. 1:89 (1794). 

Figure 4

Description. Sporophores sporocarpic, gregarious or densely crowded, mutual extrusion deformation, sessile, hemispheric to globose. Peridium is two-layered; the outer layer is limy, eggshell-like, smooth, white; the inner layer is membranous, grey-white, separate from the outer layer, iridescent. Columella is developed, hemispheric, white or pale cream, calcareous. Capillitium threads are slender, dense, pale-brown, sometimes with darker swellings, branched and anastomosed at the end, with membranous enlargement. Spores are dark in mass, reddish brown via transmitted light, with obvious warts, 9–11 μm in diam.

Habitat. On rotten branches and moss.

Distribution in China. Beijing City, Gansu Province, Hebei Province, Heilongjiang Province, Inner Mongolia Autonomous Region, Jilin Province, Liaoning Province, Shaanxi Province, and Yunnan Province.

Distribution in the world. Algeria, Australia, Austria, Canada, China, Costa Rica, Finland, France, Germany, Italy, Japan, Karelia, Korea, Latvia, Morocco, Nepal, the Netherlands, New Zealand, Norway, Peru, Russia, Spain, Sweden, Switzerland, the United States, the United Kingdom, Ukraine, and Venezuela.

Specimens examined. China, Inner Mongolia Autonomous Region, Genhe City, 207 management and protection station of forestry bureau, on the rotten branches, 4 September 2021, B. Zhang, HMJAU M20016; China, Hubei Province, Shiyan City, on the moss, 8 September 2013 B. Zhang, HMJAU M20062.

Notes. *Diderma globosum* is often confused with *D. europaeum* and *D. crustaceum* due to their similar morphology. They are all smooth, white, and hemispheric to globose sporocarp with a two-layered peridium. However, *D. globose* differs from *D. europaeum* and *D. crustaceum* due to its small spores with warts and developed and hemispheric columella.

*Diderma radiatum* (L.) Morgan, J. Cincinnati Soc. Nat. Hist. 16(4):151, 1894. 

Figure 5

Description. Fructification sporocarps, scattered, spherical or oblate, concave below the umbilicus, light yellow-brown, with floral spots and lattice concavities, 0.6–1.58 mm in diam. Sessile or short-stalked, thick, yellow-brown, sulcate, 0.2–0.6 mm in diam. Columella developed and conspicuous and then calcareous, hemispherical, orange, ivory or auburn. Peridium is two-layered; the outer layer is cartilaginous, brown, latticed; the inner layer is membranous, light reddish brown, tightly fused with the outer layer, petaloid or radially dehiscent. Hypothallus is membranous, light brown, translucent. Capillitium is thin, dense, with few branches, brown. Spores are dark in mass, purple-brown via transmitted light, globose, 9–11 μm in diam., with warts, spores accumulated surround columella, separated from the inner layer of the peridium.

Habitat. On the rotten woods.

Distribution in China. Gansu Province, Hebei Province, Henan Province, Heilongjiang Province, Inner Mongolia Autonomous Region, Jilin Province, Qinghai Province, Shaanxi Province, Taiwan Province, and Xinjiang Uygur Autonomous Region.

Distribution in the world. Australia, Austria, Argentina, Canada, China, Denmark, Finland, France, Germany, India, Italy, Japan, Karelia, New Caledonia, New Zealand, Nepal, Norway, Pakistan, Portugal, Puerto Rico, Russia, Spain, Sweden, the United States, and the United Kingdom.

Specimens examined. China, Henan Province, Sanmenxia City, Ganshan National Forest Park, on rotten wood, 26 October 2012, B. Zhang, HMJAU M20026, HMJAU M20057, HMJAU M20058, HMJAU M20059, HMJAU M20060, HMJAU M20061; China, Inner Mongolia Autonomous Region, Hinggan League, Arxan, Motianling, on rotten wood, 15 August 1985, Y. Li and S.L. Chen, HMJAU 9136; China, Inner Mongolia Autonomous Region, Genhe City, on rotten wood, 1 September 1992, W.C. Gao, HMJAU 9963.

Notes. *Diderma radiatum* is easily confused with *D. roanense* (Rex) T. Macbr. due to their highly similar morphology, such as sporocarpic appearance with sessile or short-stalked structures, two-layered peridium, large and developed columella, dehiscence floriform, and spores with warts. However, *D. radiatum* can be differentiated from *D. roanense* by its yellow-brown stalk with limy features, brown capillitium, and smaller spores.

## 4. Discussion

The *Diderma* genus is widely distributed across China, yet its complete species diversity remains unclear. This study presents comprehensive descriptions of two new species, *D*. *shaanxiense* and *D*. *clavatocolumellum*, along with two previously undocumented species, *D*. *globosum* and *D*. *radiatum*, discovered in the Inner Mongolia Autonomous Region and Henan Province. The species most closely related in micromorphology to *D. shaanxiense* appears to be *D. globosum*. *D. shaanxiense* is characterized by small, pulvinate, and shining columella, whereas *D. globosum* has a hemispherical, halo-free columella, making it easier to differentiate between these two species. *Diderma clavatocolumellum* can be readily differentiated from *D. alpinum* by its distinct features, such as a clavate columella, spores with warts, and a brown hypothallus. On the phylogenetic tree, these two species are distinctly positioned on individual branches and separate from known species, thus confirming our morphological findings. Furthermore, the phylogenetic analysis also validates certain species previously documented.

*Diderma* is divided into three main branches. The first branch, led by *D*. *effusum*, *D. verrucocapillitia*, *D. deplanatum*, *D. chondrioderma*, *D. acanthosporum*, *D. yucatanense*, *D*. *hemisphaericum*, and *D*. *pseudotestaceum*, encompasses eight species. These species are distinguished by calcareous peridium extending from the sporangium to the synangium, spores with warts, and a columella that is not distinctly pulvinate or absent. Clade 2 comprises two species, *D. niveum* and *D. alpinum*, and is characterized by a double-layered peridium, with spores mainly spiny and living in high mountain areas. In 2024, Shchepin et al. also used partial sequences of 18SrDNA to clearly distinguish these two species from some other known species [33]. The third branch consists of *D. globosum*, *D*. *europaeum*, *D. radiatum*, *D. velutinum*, *D. meyerae*, *D. floriforme*, *D. cattiense*, *D. subasteroides*, *D. gracile*, *D. tigrinum*, *D. aurantiacum*, *D.montanum*, *D. umbilicatum*, *D. microcarpum*, *D. kamchaticum*, and two newly discovered species in this study, *D. shaanxiense* and *D. clavatocolumellum*, totaling seventeen species, and the main feature of this branch is that the columella is more prominent, hemispherical or clavate, and the spores are mainly warty.

In the past, morphology was predominantly used for classification and identification purposes. However, molecular barcoding and phylogeny have emerged as a reliable tool for verifying species. These approaches indicate that the species diversity of myxomycetes is not yet fully understood [34]. Phylogenetic trees constructed by incorporating molecular barcodes into numerous morphologically described species have revealed that these species are often complex, comprising several or even dozens of distinct species [6,21,35,36,37,38,39]. 

In order to evaluate the compatibility of the marker sequences, the partition homogeneity test was performed. The results indicated sufficient congruence among the marker sequences, justifying their concatenation. The balanced index values for the concatenated dataset suggest a robust and reliable phylogenetic inference, minimizing biases from individual markers. In our constructed phylogenetic tree, *Physarum* Pers., *Badhamia* Berk., and *Fuligo* Haller are sister branches, suggesting that the taxonomic status of *Physarum* and *Badhamia* is uncertain and that they may be polyphyletic. This finding is consistent with previous studies by Nandipati et al. [40], Cainelli et al. [41], Prikhodko et al. [7], and Ronikier et al. [42]. Prikhodko et al. [7] conducted a phylogenetic analysis of species within *Didymiaceae* (Myxomycetes) based on three independent genetic markers (18S rDNA gene, translation elongation factor 1-alpha, and cytochrome c oxidase), which supported taxonomic rearrangements in this family. Their study revealed that all studied species of *Lepidoderma* formed a distinct clade along with *Diderma fallax*, suggesting that they should be classified under the genus *Polyschismium*. Additionally, *Lepidoderma tigrinum* and two related species were transferred to the genus *Diderma*, leading to the invalidation of the genus *Lepidoderma* and an increase in species diversity within the genus *Diderma*.

The current study has expanded the known species diversity of the genus *Diderma* in China. However, ongoing debates persist regarding the taxonomic classification of this genus, largely due to limited species sampling and insufficient genetic variation in DNA loci. Therefore, additional evidence is required to enhance our understanding of this genus comprehensively. Despite recent discoveries of new *Diderma* species in China, the full extent of its species diversity remains uncertain, highlighting the need for a comprehensive systematic analysis.

## Figures and Tables

**Figure 2:**
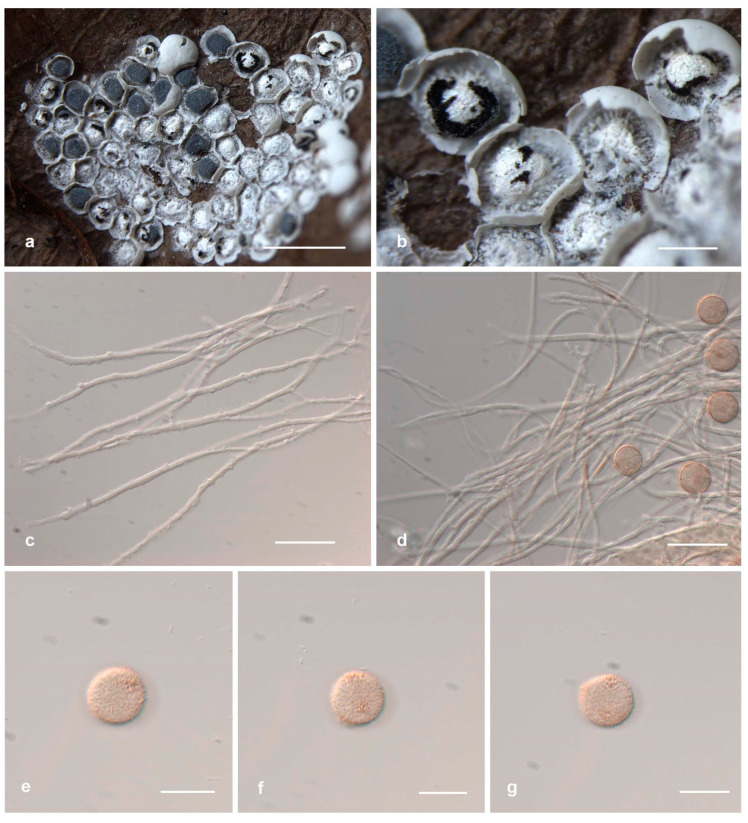
Habitat and microstructure of *Diderma shaanxiense* (HMJAU M20033 Holotype). (**a**,**b**) Sporangia and columella; (**c**,**d**) capillitium and some spores via TL; (**e**–**g**) spores via TL. Scale bars: (**a**) = 2 mm, (**b**) = 500 μm, (**c**,**d**) = 20 μm, (**e**–**g**) = 10 μm.

**Figure 3:**
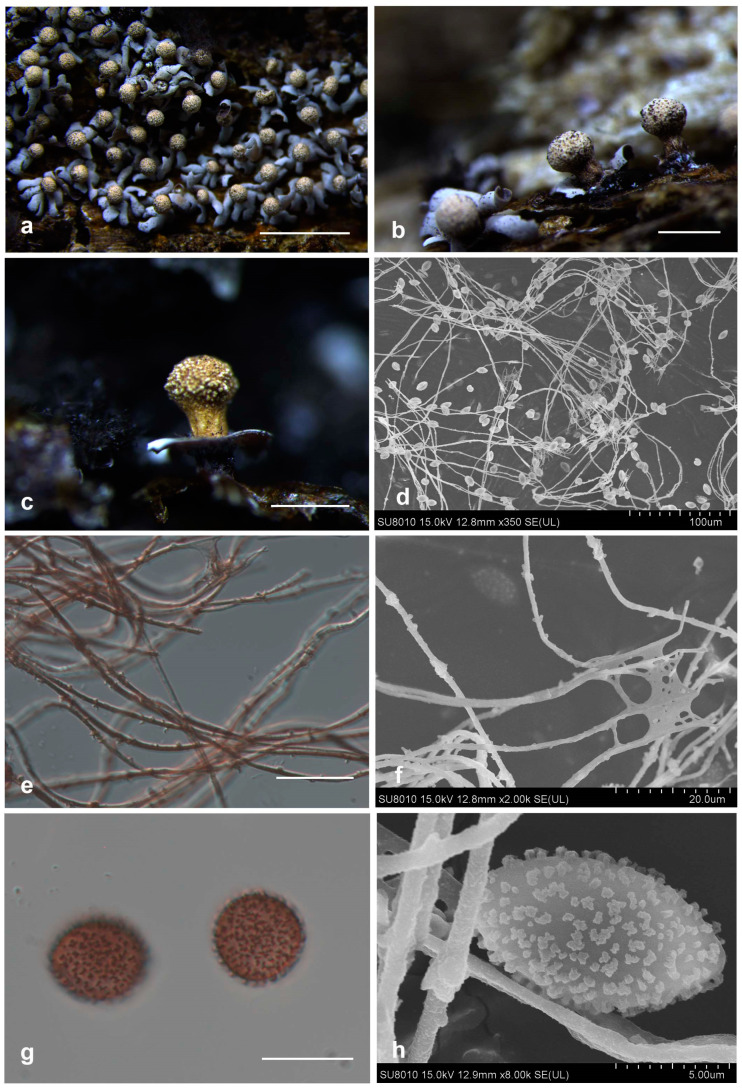
Habitat and microstructure of *Diderma clavatocolumellum* (HMJAU 11,084 Holotype). (**a**,**b**) Sporocarpous and columella; (**c**) columella and short-stalk sporocarpic; (**d**) capillitium and some spores via SEM; (**e**,**f**) capillitium with many dark-brown swollen knots via TL and SEM; (**g**,**h**) spores with warts and ridges via TL and SEM. Scale bars: (**a**) = 2 mm, (**b**,**c**) = 500 mm, (**e**,**g**) = 10 μm.

**Figure 4:**
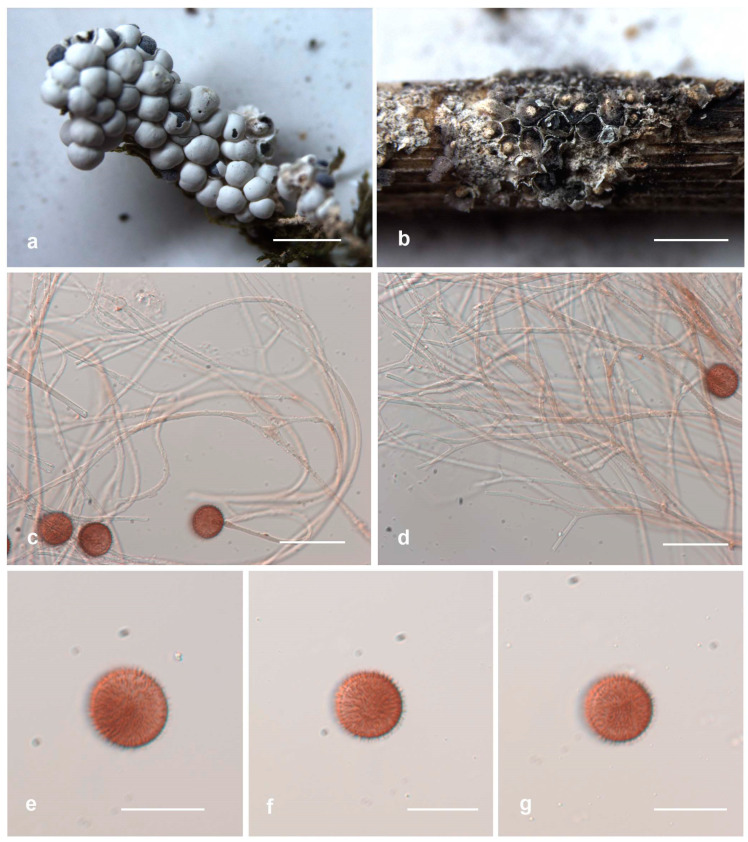
Habitat and microstructure of *Diderma globosum* (HMJAU M20016). (**a**) Sporocarpous; (**b**) columella; (**c**,**d**) capillitium and spores via TL; (**e**–**g**) spores with verrucose via TL. Scale bars: (**a**,**b**) = 2 mm, (**c**,**d**) = 20 μm, (**e**–**g**) = 10 μm.

**Figure 5:**
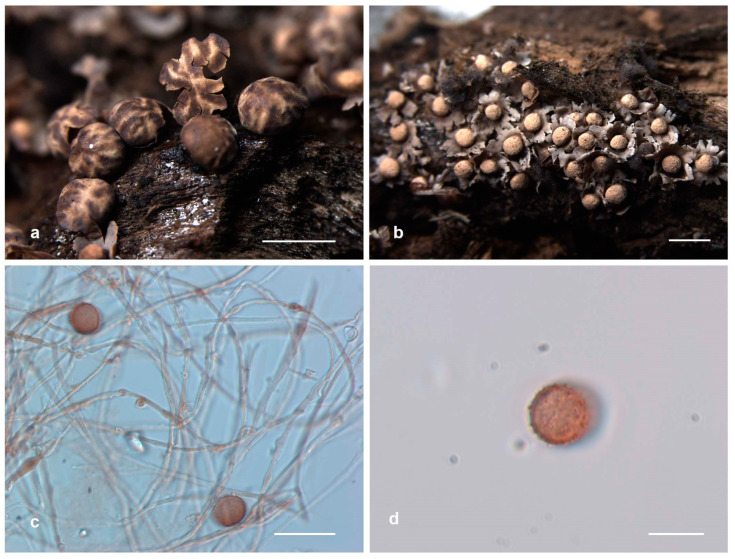
Habitat and microstructure of *Diderma radiatum* (HMJAU M20026). (**a**) Sporocarpous; (**b**) columella; (**c**) capillitium and spores via TL; (**d**) Spores with verrucose via TL. Scale bars: (**a**,**b**) = 2 mm, (**c**) = 20 μm, (**d**) = 10 μm.

**Table 1:** Information for the sequences produced in this study.

Scientific Name	Voucher/Specimen Numbers	GenBank Accession Numbers			
		nSSU	EF-1α	COI	
*D. clavatocolumellum*	HMJAU 11084-1	PP165417	PP178585	PP261313	This study
*D. clavatocolumellum*	HMJAU 11084-2	PP165418	PP982177	PP261314	This study
*D. globosum*	HMJAU M20016	PP165444	PP178604		This study
*D. radiatum*	HMJAU M20026-1	PP165459	PP178614	PP261321	This study
*D. radiatum*	HMJAU M20026-2	PP165460	PP178615	PP261322	This study
*D.* *shaanxiense*	HMJAU M20033	PP165469	PP178624	PP261324	This study
*D.* *shaanxiense*	HMJAU M20034	PP165470	PP982178	PP261325	This study

## Data Availability

The original contributions presented in the study are included in the article/Supplementary Material, further inquiries can be directed to the corresponding author/s.

## Supplementary Materials

The following supporting information can be downloaded at: https://www.mdpi.com/article/10.3390/jof10080514/s1, Table S1: Information for the sequences from database used in this study [43,44,45,46,47,48,49,50,51,52,53,54]; Table S2: Partition homogeneity test.

## References

[1] Stephenson S.L., Schnittler M., Novozhilov Y.K. (2008). Myxomycete diversity and distribution from the fossil record to the present. Biodivers. Conserv..

[2] Adl S.M., Bass D., Lane C.E., Lukeš J., Schoch C.L., Smirnov A., Agatha S., Berney C., Brown M.W., Burki F. (2019). Revisions to the classification, nomenclature, and diversity of eukaryotes. J. Eukaryot. Microbiol..

[3] Martin G.W., Alexopoulos C.J. (1969). The Myxomycetes. The Myxomycètes.

[4] García-Martín J.M., Zamora J.C., Lado C. (2023). Multigene phylogeny of the order Physarales (Myxomycetes, Amoebozoa): Shedding light on the dark-spored clade. Persoonia.

[5] Nmf D. (1794). Neues Magazin für die Botanik in Ihrem Ganzen Umfange: Band 1.

[6] Leontyev D.V., Schnittler M., Stephenson S.L., Novozhilov Y.K., Shchepin O.N. (2019). Towards a phylogenetic classification of the Myxomycetes. Phytotaxa.

[7] Prikhodko I.S., Shchepin O.N., Bortnikova N.A., Novozhilov Y.K., Gmoshinskiy V.I., Moreno G., López-Villalba N., Stephenson S.L., Schnittler M. (2023). A three-gene phylogeny supports taxonomic rearrangements in the family *Didymiaceae* (Myxomycetes). Mycol Prog..

[8] Lado C., Eliasson U., Rojas C., Stephenson S.L. (2022). Taxonomy and systematics: Current knowledge and approaches on the taxonomic treatment of Myxomycetes: Updated version. Myxomycetes.

[9] Rostafiński J.T. Versuch Eines Systems der Mycetozoen. F. Wolff 1873, 9–13. https://BiotaNZ.landcareresearch.co.nz/references/1cb0f8b9-36b9-11d5-9548-00d0592d548c.

[10] Chow Z.H. (1978). Taxonomic information of Myxomycetes. Jilin Agricultural University, Institute of Microbiology.

[11] Lado C. (2005–2024) An Online Nomenclatural Information System of Eumycezotozoa. http://www.nomen.eumycetozoa.com.

[12] Song W.L., Lin D., Li M., Du Q., Chen S.L. (2022). Four new records of myxomycetes from China. Phytotaxa.

[13] Gao Y., Yan S.Z., Wang G.W., Chen S.L. (2018). Two new species and two new records of myxomycetes from subtropical forests in China. Phytotaxa.

[14] Zhao H.N., Rao G., Yang X.Y., Li X., Yu H.L., Zhang B., Li Y. (2022). Two new species of *Diderma* (Physarales, *Didymiaceae*) from northern China. Phytotaxa.

[15] Chen S.L., Yan S.Z., Li Y. (2013). Species of the genus *Diderma* from China and their distributions. Mycosystema.

[16] Liu C.H., Chang J.H. (2011). Myxomycetes of Taiwan XXI. The genus *Diderma*. Taiwania.

[17] Royal B.G. (1969). Edinburgh. Flora of British Fungi: Colour Identification Chart.

[18] Wang W., Wang W., Wei S.W., Huang W., Qi B., Wang Q., Li Y. (2021). Design of potentially universal SSU primers in myxomycetes using next-generation sequencing. J. Microbiol. Methods.

[19] Novozhilov Y.K., Okun M.V., Erastova D.A., Shchepin O.N., Zemlyanskaya I.V., García-Carvajal E., Schnittler M. (2013). Description, culture and phylogenetic position of a new xerotolerant species of *Physarum*. Mycologia.

[20] Liu Q.S., Yan S.Z., Chen S.L. (2015). Further resolving the phylogeny of Myxogastria (slime molds) based on COI and SSU rRNA genes. Russ. J. Genet..

[21] Feng Y., Schnittler M. (2015). Sex or no sex? Group I introns and independent marker genes reveal the existence of three sexual but reproductively isolated biospecies in *Trichia varia* (Myxomycetes). Org. Divers. Evol..

[22] Novozhilov Y.K., Prikhodko I.S., Shchepin O.N. (2019). A new species of *Diderma* from Bidoup Nui Ba National Park (southern Vietnam). Protistology.

[23] Standley D.M. (2013). MAFFT multiple sequence alignment software version 7: Improvements in performance and usability. Mol. Biol. Evol..

[24] Thompson J.D., Gibson T.J., Plewniak F. (1997). The Clustal–X windows interface: Flexible strategies for multiple sequence alignment aided by quality analysis tools. Nucleic Acids Res..

[25] Hall T. (1999). BioEdit: A user-friendly biological sequence alignment editor and analysis program for Windows 95/98/NT. Nucleic Acids Symp. Ser..

[26] Goloboff P.A., Catalano S.A., Torres A. (2022). Parsimony analysis of phylogenomic datasets (II): Evaluation of PAUP*, MEGA and MPBoot. Cladistics.

[27] Kalyaanamoorthy S., Minh B.Q., Wong T.K.F., Haeseler A.V., Jermiin L.S. (2017). ModelFinder: Fast model selection for accurate phylogenetic estimates. Nat. Methods..

[28] Nguyen L.T., Schmidt H.A., Haeseler A.V., Minh B.Q. (2015). IQ-TREE: A fast and effective stochastic algorithm for estimating maximum-likelihood phylogenies. Mol. Biol. Evol..

[29] Zhaxybayeva O., Gogarten J.P. (2002). Bootstrap, Bayesian prob-ability and maximum likelihood mapping: Exploring newtools for comparative genome analyses. BMC Genom..

[30] Huelsenbeck J.P., Ronquist F. (2001). MRBAYES: Bayesian inference of phylogenetic trees. Bioinformatics.

[31] Rambaut A., Drummond A.J., Xie D., Baele G., Suchard M.A. (2018). Posterior Summarization in Bayesian Phylogenetics Using Tracer 1.7. Syst Biol..

[32] Bork P. (2007). Interactive Tree of Life (iTOL): An online tool for phylogenetic tree display and annotation. Bioinformatics.

[33] Shchepin O.N., López V.Á., Inoue M., Prikhodko I.S., Erastova D.A., Okun M.V., Woyzichovski J., Yajima Y., Gmoshinskiy V.I., Moreno G. (2024). DNA barcodes reliably differentiate between nivicolous species of *Diderma* (Myxomycetes, Amoebozoa) and reveal regional differences within Eurasia. Protist.

[34] Leontyev D.V., Schnittler M., Rojas Alvarado C., Stephenson S.L. (2022). The phylogeny and phylogenetically based classification of myxomycetes. Myxomycetes: Biology, Systematics, Biogeography and Ecology.

[35] Shchepin O., Novozhilov Y., Woyzichovski J., Bog M., Prikhodko I., Fedorova N., Gmoshinskiy V., Borg D.M., Dagamac N.H.A., Yajima Y. (2022). Genetic structure of the protist *Physarum albescens* (Amoebozoa) revealed by multiple markers and genotyping by sequencing. Mol. Ecol..

[36] Dagamac N.H.A., Rojas C., Novozhilov Y.K., Moreno G.H., Schlueter R., Schnittler M. (2017). Speciation in progress? A phylogeographic study among populations of *Hemitrichia serpula* (Myxomycetes). PLoS ONE.

[37] Leontyev D.V., Schnittler M., Stephenson S.L. (2015). A critical revision of the *Tubifera ferruginosa*-complex. Mycologia.

[38] Leontyev D.V., Buttgereit M., Kochergina A., Shchepin O.N., Schnittler M. (2023). Two independent genetic markers support separation of the myxomycete *Lycogala epidendrum* into numerous biological species. Mycologia.

[39] Leontyev D.V., Ishchenko Y., Schnittler M. (2023). Fifteen new species from the genus *Lycogala* (Myxomycetes). Mycologia.

[40] Nandipati S.C., Haugli K., Coucheron D.H., Haskins E.F., Johansen S.D. (2012). Polyphyletic origin of the genus *Physarum* (Physarales, Myxomycetes) revealed by nuclear rDNA mini-chromosome analysis and group I intron synapomorphy. BMC Evol. Biol..

[41] Cainelli R., Haan M., Meyer M., Bonkowski M., Fiore-Donno A.M. (2020). Phylogeny of Physarida (Amoebozoa, Myxogastria) based on the small-subunit ribosomal RNA gene, redefinition of *Physarum pusillum* s. str. and reinstatement of *P. gravidum* Morgan. J. Eukaryot Microbiol..

[42] Ronikier A., Janik P., Haan M., Kuhnt A., Zankowicz M. (2022). Importance of type specimen study for understanding genus boundaries-taxonomic clarifications in *Lepidoderma* based on integrative taxonomy approach leading to resurrection of the old genus *Polyschismium*. Mycologia.

[43] García-Martín J.M., Mosquera J., Lado C. (2018). Morphological and molecular characterization of a new succulenticolous *Physarum* (Myxomycetes, Amoebozoa) with unique polygonal spores linked in chains. Eur. J. Protistol..

[44] Gmoshinskiy V.I., Prikhodko I.S., Bortnikov F.M., Shchepin O.N., Schnittler M. (2023). Morphology and phylogeny of *Diderma aurantiacum* (myxomycetes)-A new species for Russia from the far east. Hobocmu Cucemamuku Hu3wux Oacmehuu.

[45] Novozhilov Y.K., Mitchell D.W., Okun M.V., Shchepin O.N. (2014). New species of *Diderma* from Vietnam. Mycosphere.

[46] Hoppe T., Schnittler M. (2015). Characterization of myxomycetes in two different soils by TRFLP- analysis of partial 18S rRNA gene sequences. Mushroom Research Foundation. Mycosphere.

[47] Novozhilov Y.K., Shchepin O.N., Prikhodko I., Schnittler M. (2022). A new nivicolous species of *Diderma* (Myxomycetes) from Kamchatka, Russia. Nova Hedwig. Z. Fur Kryptogamenkd..

[48] Bortnikov F.M., Shchepin O.N., Gmoshinskiy V.I., Prikhodko I.S., Novozhilov Y.K. (2018). *Diderma velutinum*, a new species of *Diderma* (Myxomycetes) with large columella and triple peridium from Russia. Bot. Pacifica.

[49] Lado C., Treviño Z.I., García-Martín J.M., Basanta D.W. (2022). *Diachea mitchellii*: A new myxomycete species from high elevation forests in the tropical Andes of Peru. Mycologia.

[50] Wikmark O.G., Haugen P., Lundblad E.W., Haugli K., Johansen S.D. (2007). The molecular evolution and structural organization of group I introns at position 1389 in nuclear small subunit rDNA of myxomycetes. J. Eukaryot Microbiol..

[51] Janik P., Lado C., Ronikier A. (2020). Range-wide Phylogeography of a nivicolous protist *Didymium nivicola* Meyl. (Myxomycetes, Amoebozoa): Striking contrasts between the northern and the southern hemisphere. Protist.

[52] Zhao F.Y., Liu S.Y., Stephenson S.L., Hsiang T., Qi B., Li Z. (2021). Morphological and molecular characterization of the new aethaloid species *Didymium yulii*. Mycologia.

[53] Fiore-Donno A.M., Berney C., Pawlowski J., Baldauf S.L. (2005). higher-order phylogeny of plasmodial slime molds (Myxogastria) based on elongation factor 1-A and small subunit rRNA gene sequences. J. Eukaryot Microbiol..

[54] Borg D.M., Brejnrod A.D., Unterseher M., Hoppe T., Feng Y., Novozhilov Y., Sørensen S.J., Schnittler M. (2018). Genetic barcoding of dark-spored myxomycetes (Amoebozoa)-Identification, evaluation and application of a sequence similarity threshold for species differentiation in NGS studies. Mol. Ecol. Resour..

