# Supplementary material for: Two New Species of the Genus Diderma (Physarales, Didymiaceae) in China with an Addition to the Distribution
Source: J Fungi (Basel). 2024 Jul 23;10(8):514. doi: 10.3390/jof10080514 (PMC11355789; doi:10.3390/jof10080514)
Supplement: Supplementary file 1 [file jof-10-00514-s001.zip › jof-3067087-supplementary.pdf]

Table S1. Information for the sequences from database used in this study.

Scientific name	Voucher/specimen numbers	GenBank accession numbers			Reference
		nSSU	EF-1α	COI	
Badhamia melanospora	MA-Fungi 81706	KC758975	MG963469		[4]
B. melanospora	MA-Fungi 88015	MF352450	MF352496		[43]
B. melanospora	MA-Fungi 64653	OP650733	MG963468		[4]
B. melanospora	MA-Fungi 80423	MF352448	MF352494		[32]
D. acanthosporum	MA-Fungi 90993	MG963648	MG963480		[4]
D. acanthosporum	MA-Fungi 90923	MG963647	MG963479		[4]
Diderma alpinum	sc30044	MN595463	MN596920		[33]
D. alpinum	YK385	MN595608	OP616431	OP616538	[7]
D. aurantiacum	LE286478	OQ312103	OQ320534	OQ320546	[44]
D. aurantiacum	LE302633	OQ312104	OQ320535	OQ320547	[44]
D. cattiense	LE291341	KJ659865	KJ676603		[45]
D. cattiense	LE286673	KJ659863	KJ676601		[45]
D. chondrioderma	MYX439	KM977850	MK555282		[46]
D. deplanatum	MYX440	KM977851	MK555283		[46]
D. floriforme	MdH1810007	OM339175			[42]
D. floriforme	MYX18793	OP621222	OP616432	OP616547	[7]
D. globosum	LE325799	MZ604992	MZ605421	OP616551	[47]
D. globosum	LE325800	MZ604993	MZ605422	OP616552	[47]
D. globosum	LE325793	MZ604991	MZ605420	OP616550	[47]
D. globosum	LE325710	MZ604990	MZ605419	OP616549	[47]
D. globosum	LE325132	MZ604989	MZ605418	OP616548	[47]
D. effusum	MYX7994	MZ604987	MZ605416	OP616543	[47]
D. europaeum	MM38936	PP099459		PP099459	[33]
D. europaeum	MA-Fungi 80025	MG963658	MG963489		[4]
D. europaeum	LE289737	KR024552	KU198104		unpublished
D. europaeum	sc30832		PP097317		[33]
D. europaeum	MA-Fungi 73109	MG963656	MG963487		[4]
D. europaeum	MA-Fungi 73112	MG963657	MG963488		[4]
D. gracile	MA-Fungi 83291	MW240309	MW240039		[4]
D. gracile	MA-Fungi 78767		MW240038		[4]
D. hemisphaericum	MA-Fungi 63960	MG963659	MG963490		[4]
D. hemisphaericum	MA-Fungi 90974	MG963660	MG963491		[4]
D. hemisphaericum	MA-Fungi 91235	MW240310			[4]
D. hemisphaericum	MA-Fungi 91206	MG963661	MG963492		[4]
D. kamchaticum	sc29935	MN595440	MN596915		[33]
D. kamchaticum	sc29898	MN595425	MN596914		[33]
D. meyerae	LE284739	KU198050	KU198109		unpublished
D. meyerae	sc30279	MN595488	MN596919		[33]
D. microcarpum	LE289852	KR029679	KU198128		unpublished
D. microcarpum	LE289848	KR029678	KU198127		unpublished

D. montanum	MYX8967	OQ312106	OQ320537	PP097348	[44]
D. montanum	MYX8966	OQ312105	OQ320536	PP097346	[44]
D. niveum	MA-Fungi 78779	MW240312	MW240041		[4]
D. niveum	MA-Fungi 78780	MW240313			[4]
D. pseudotestaceum	LE291396	KJ659866	KJ676604		[45]
D. pseudotestaceum	LE291397	KJ659867	KJ676605		[45]
D. pseudotestaceum	LE291398	KJ659864	KJ676602		[45]
D. radiatum	MYX9867	MZ604983	MZ605412	OP616561	[7] [47]
D. radiatum	MYX9071	MK838444	MZ605410	OP616560	[7] [47]
D. radiatum	MYX9084	MK838445	MZ605411		[47]
D. subasteroides	MA-Fungi 91218		MW240043		[4]
D. tigrinum	MA-Fungi 81004	MW240363			[4]
D. tigrinum	MA-Fungi 52983	MW240362			[4]
D. umbilicatum	MYX8785	OQ312114	OQ320545	PP097345	[44]
D. umbilicatum	MYX15083	OQ312113	OQ320544		[44]
D. velutinum	LE318752	MH714785	MH717084	MH71708<br>6	[48]
D. velutinum	LE318753	MH714786	MH717085	MH71708<br>7	[48]
D. verrucocapillitia	HMJAU 60258	OM258684			[14]
D. yucatanense	MA-Fungi 51222		ON081613		[49]
Didymium dubium	MA-Fungi 63904	MW240326	MW240058		[4]
D. dubium	MA-Fungi 80036	MW240327	MW240059		[4]
D. dubium	MA-Fungi 80492	MG662512	MW240060		[4]
D. dubium	K7	AM231294			[50]
D. melanospermum	MA-Fungi 91238	MG963668	MG963497		[4]
D. melanospermum	MA-Fungi 62790	MG963667	MW240068		[4]
D. nivicola	MA-Fungi 90573	MT227090	MT230925		[51]
D. nivicola	AH19667	MT227019	MT230908		[51]
D. pseudonivicola	MA-Fungi 90601	MT227112	MT230931		[51]
D. pseudonivicola	MA-Fungi 90587	MT227099	MT230927		[51]
D. yulii	HMJAU M3002	MF149871	MK905755		[52]
D. yulii	HMJAU M3001	MF149870	MK905754		[52]
Echinostelium coelocephalum	ATCC MYA 2964	AY842033	AY643813		[53]
E. minutum	ATCC 22345	AY842034	AY643814		[53]
Fuligo septica	MA-Fungi 78118	MW240350	MW240091		[4]
F. septica	MA-Fungi 78792	MF352458	MF352504		[43]
F. septica	MA-Fungi 78801	MF352459	MF352505		[43]
Physarum didermoides	MA-Fungi 71195	MW240378			[4]
P. didermoides	MA-Fungi 57262	MF352488	MF352542		[43]
P. leucophaeum	MA-Fungi 49730	MG963686	MG963521		[4]
P. leucophaeum	MA-Fungi 78861	MG963688			[4]

P. leucophaeum	MA-Fungi 59323	MF352477	MF352526		[43]
P. nivale	MA-Fungi 72831	MF352486	MF352536		[43]
P. nivale	MA-Fungi 73457	MF352487	MF352537		[43]
P. nivale	MA-Fungi 70191	MW240384	MG963529		[4]
P. nivale	MA-Fungi 70193	MW240385			[4]
P. pseudonotabiles	LE255432	LT670439	KF250465		[54]
P. pseudonotabiles	LE255437	LT670419	KC473813		[45] [54]
P. pseudonotabiles	LE255703	LT670568	KF250468		[54]
P. pseudonotabiles	LE284662	LT670428	KF250472		[54]
P. straminipes	MA-Fungi 70363	MF352489	MF352543		[43]
P. straminipes	MA-Fungi 87865	MW240394			[4]
P. viride	LE302489	MW693022	MW701670		[36]
P. viride	LE317322	MW693024	MW701672	OP616654	[7][36]

Table S2 Partition homogeneity test

**Genes**	**Tree**	**Length**	**Consistency index (C.I.)**	**Retention index (R.I.)**	**Rescaled consistency index (R.C.)**	**Homogeneity index（H.I. ）**
SSU	1	2947	0.547336	0.771849	0.422461	0.452664
EF-1A	1	2130	0.477934	0.720954	0.344568	0.522066
COI	1	1013	0.556762	0.676746	0.376786	0.443238
SSU+COI	1	3146	0.502861	0.706015	0.355027	0.497139
SSU+EF1A+COI	1	6174	0.517817	0.733602	0.379871	0.482183
